# Opinions towards Aging in the Marshallese Community residing in Northwest Arkansas

**DOI:** 10.1093/geroni/igaf122.2209

**Published:** 2025-12-31

**Authors:** Shakshi Sharma, Gohar Azhar, Karen Coker, Sheldon Riklon, Philmar Kabua, Pearl Mcelfish, Jeanne Wei

**Affiliations:** University of Arkansas for Medical Sciences, Little Rock, Arkansas, United States; University of Arkansas for Medical Sciences, Little Rock, AR, LITTLE ROCK, Arkansas, United States; University of Arkansas for Medical Sciences, Department of Geriatrics, Donald W. Reynolds institute of Aging, 4301 West Markham Street, Slot 748, little Rock, AR, 72205-7199, USA, Little Rock, Arkansas, United States; University of Arkansas for Medical Sciences Northwest, Fayetteville, Arkansas, United States; University of Arkansas for Medical Sciences Northwest, Fayetteville, Arkansas, United States; University of Arkansas for Medical Sciences Northwest, Fayetteville, Arkansas, United States; University of Arkansas for Medical Sciences, Little Rock, AR, LITTLE ROCK, Arkansas, United States

## Abstract

The Marshallese is a Pacific Islander community located around the US with much of the population primarily residing in Northwest Arkansas. The perception of aging in this community might be influenced by different cultural traditions, health conditions as well as health information and education. We sought to develop an understanding of how Marshallese view aging. An anonymous survey exploring perspectives and beliefs regarding aging was administered to fifty subjects (mean age 46.7±11.1 SD). This was a convenience sample with 68% of the participants between 18-50 yrs. and 32% between 51-89, with 72% females and 28% males. When we asked participants if aging was a natural process, 86% agreed, 6% disagreed and 8% were uncertain. Furthermore 52% agreed when asked if getting older was associated with fewer activities, 22% disagreed and 26% were uncertain. When asked if aging was a possible punishment or sin, 18% agreed, 59% disagreed and 22% were uncertain. Additionally, 12 % believed that Alzheimer’s disease was a punishment for sin. Although the majority of our participants were younger adults, only 38% believed they were young, whereas 32% classified themselves as being “old” and 30% were uncertain. The results from our survey highlighted the somewhat unfavorable beliefs of Marshallese towards aging and dementia. Understanding these perspectives in the context of their traditions and culture will assist with developing educational materials to promote a healthier and more positive image of aging while improving the quality of life of the older adult Marshallese community members.

